# Diagnostic Reclassification From Parkinson’s Disease to Multiple System Atrophy Based on Longitudinal Clinical and Imaging Findings: A Case Report

**DOI:** 10.7759/cureus.110346

**Published:** 2026-06-06

**Authors:** Shoji Kikui, Yoshihiro Kashiwaya, Daisuke Danno, Takao Takeshima

**Affiliations:** 1 Neurology, Tominaga Hospital, Osaka, JPN

**Keywords:** 123i-mibg myocardial scintigraphy, diagnostic approach, longitudinal multimodal diagnostic approach, multiple system atrophy (msa), parkinson's disease, selegiline

## Abstract

It is often challenging to differentiate Parkinson’s disease (PD) from multiple system atrophy (MSA) - particularly, the parkinsonian subtype (MSA-P) - in the early disease stages because of overlapping clinical features. Here, we report the case of a 72-year-old man who was initially diagnosed with PD but whose diagnosis was later changed to MSA-P following the emergence of autonomic dysfunction and characteristic magnetic resonance imaging (MRI) findings. He presented with tremors, rigidity, bradykinesia, and postural instability, and dopamine transporter imaging demonstrated bilateral presynaptic dopaminergic dysfunction. Cardiac ^123^I-metaiodobenzylguanidine (MIBG) scintigraphy revealed reduced uptake on initial evaluation, which was considered supportive of PD. However, the response to levodopa was limited. During follow-up, several atypical features became apparent, including Pisa syndrome, early postural instability, orthostatic hypotension, and neurogenic bladder. Follow-up MRI revealed a hyperintense rim along the lateral margins of the bilateral putamina, suggesting MSA-P; these findings provided a key objective basis for the diagnostic reclassification. Repeat MIBG scintigraphy performed after selegiline discontinuation revealed an improvement in the heart-to-mediastinum ratio, suggesting a possible drug-related effect on tracer uptake. This case illustrates the limitations of single-time-point diagnostic assessments and highlights the value of longitudinal, multimodal evaluation. It also suggests that MIBG abnormalities may be reversible, underscoring the need for cautious interpretation of MIBG findings in the differential diagnosis of PD and MSA.

## Introduction

Parkinson’s disease (PD) and multiple system atrophy (MSA) are progressive neurodegenerative disorders that can present with parkinsonism. Their clinical overlap is particularly prominent in the early stages, making differentiation challenging. Among the MSA subtypes, MSA with predominant parkinsonism (MSA-P) often mimics PD and may therefore be initially misdiagnosed [1]. Accurate differentiation between PD and MSA is clinically important because MSA generally progresses more rapidly than PD, is more frequently associated with prominent autonomic dysfunction, and typically shows a limited and less sustained response to levodopa [1,2]. Early recognition of these differences is essential for prognostic counseling, management of autonomic complications, and appropriate planning of supportive care. Recently updated diagnostic criteria for MSA have emphasized the importance of a comprehensive assessment that integrates clinical features with supportive investigations to improve diagnostic accuracy [2].

Iodine-123 metaiodobenzylguanidine (^123^I-MIBG) has been widely used as a supportive tool to distinguish PD from atypical parkinsonian syndromes. Reduced cardiac MIBG uptake is typically observed in patients with PD because of degeneration of postganglionic sympathetic nerve terminals, whereas cardiac MIBG uptake is relatively preserved in patients with MSA [3-5]. However, MIBG scintigraphy is not a definitive diagnostic test, and atypical or overlapping findings may occur in individual cases, potentially reducing its diagnostic specificity [6]. Therefore, MIBG findings should be interpreted as part of a comprehensive diagnostic assessment rather than in isolation.

Magnetic resonance imaging (MRI) also plays an important role in the differential diagnosis of MSA-P and PD. Characteristic MRI findings in MSA-P include putaminal abnormalities, such as a hyperintense rim along the lateral margin of the putamen, putaminal atrophy, hypointense putaminal signal changes, and diffusion abnormalities. Other supportive MRI features of MSA include pontocerebellar atrophy and the hot cross bun sign. Advances in imaging techniques have improved the ability to distinguish MSA from PD through quantitative assessments [2,7,8].

The accurate differentiation between PD and MSA therefore requires a multimodal approach that integrates clinical findings with both functional and structural imaging. Herein, we report a case initially diagnosed with PD but subsequently diagnosed with MSA-P based on clinical progression and evolving imaging findings. This case highlights the limitations of single-time-point evaluations and underscores the importance of longitudinal assessments in clinical practice.

## Case presentation

A 72-year-old Japanese man presented with progressive gait disturbance. His medical history was notable for hyposmia and severe constipation. The patient had no significant family history. He was a non-smoker and did not consume alcohol.

In March 2017, he began to notice difficulty walking and was diagnosed with parkinsonism at a local clinic. Levodopa/carbidopa was initiated at 100 mg/day, but no clear clinical benefit was observed. In May, i.e., after two months, tremors of the left hand developed, and he was referred to another neurology department five months later, i.e., October. Neurological examination revealed rigidity, tremors, and bradykinesia. Brain MRI and magnetic resonance angiography revealed no abnormalities, and he was diagnosed with PD. Temporary discontinuation of levodopa/carbidopa worsened the symptoms, and the medication was resumed and gradually increased to 200 mg/day.

At his first visit to our department after 16 months, i.e., in July 2018, he exhibited rigidity, tremor, and bradykinesia, predominantly affecting the left upper and lower extremities, as well as postural instability. His Hoehn and Yahr stage was three [9]. Levodopa/carbidopa was increased to 300 mg/day, and selegiline 2.5 mg/day was added; however, no marked improvement was observed. He was admitted in late September for medication adjustment and rehabilitation.

On admission, his height was 161 cm and weight was 61 kg. His blood pressure was 116/69 mmHg, and his pulse was 77 beats per minute and regular. Physical examination of the chest and abdomen was unremarkable, and there was no evidence of peripheral edema. Neurologically, he was alert. He presented with masked facies and moderate dysarthria. Rigidity was severe in the neck and right extremities and moderate in the left extremities. Resting tremors were present in both upper limbs. Finger tapping and pronation-supination movements were moderately to severely impaired, whereas lower limb tapping was mildly to moderately impaired. Pisa syndrome with rightward trunk deviation, stooped posture, short-stepped gait, and reduced arm swing was observed. Deep tendon reflexes were normal, and no pathological reflexes were observed. No muscle weakness, ataxia, or sensory disturbances were present. Although constipation was noted, no urinary dysfunction or orthostatic hypotension was observed.

Laboratory tests, including complete blood count, biochemical analysis, thyroid function tests, and urinalysis, were within normal limits. Laboratory findings on admission are shown in Table 1.

**Table 1:** Laboratory findings on admission RBC: red blood cell count; Hct: hematocrit; WBC: white blood cell count; PLT: platelet count; AST: aspartate aminotransferase; ALT: alanine aminotransferase; LDH: lactate dehydrogenase; γ-GT: gamma-glutamyl transferase; BUN: blood urea nitrogen; CRE: creatinine; GLU: glucose; CK: creatine kinase; CRP: C-reactive protein; TSH: thyroid-stimulating hormone; fT4: free thyroxine; K: Potassium; Cl: Chloride.

Parameter	At admission	Reference range
Blood test		
RBC (×10^4^/μL)	440	380-480
Ht (%)	41.5	35.0-47.0
WBC (/μL)	5,550	4,000-8,000
PLT (×10^4^/μL)	21.4	14.0-35.0
AST (IU/L)	14	10-40
ALT (IU/L)	16	5-40
LDH (IU/L)	141	115-245
γ-GT (IU/L)	22	0-30
BUN (mg/dL)	13.4	6.0-20.0
CRE (mg/dL)	0.7	0.4-0.8
GLU (mg/dL)	90	70-110
CK (IU/L)	59	32-180
Na (mEq/L)	142	137-148
K (mEq/L)	3.8	3.6-5.0
Cl (mEq/L)	105	98-109
CRP (mg/dL)	0.02	<0.30
TSH (μIU/mL)	4.40	0.54-5.54
fT4 (ng/dL)	1.60	0.97-1.72
Urinalysis		
Protein (mg/dL)	0	0-16
Glucose (mg/dL)	0	0-25
Occult blood (mg/dL)	0	0-0.01
Ketone bodies (mg/dL)	0	0-0.01
pH	6.0	4.5-8.0

**Figure 1:**
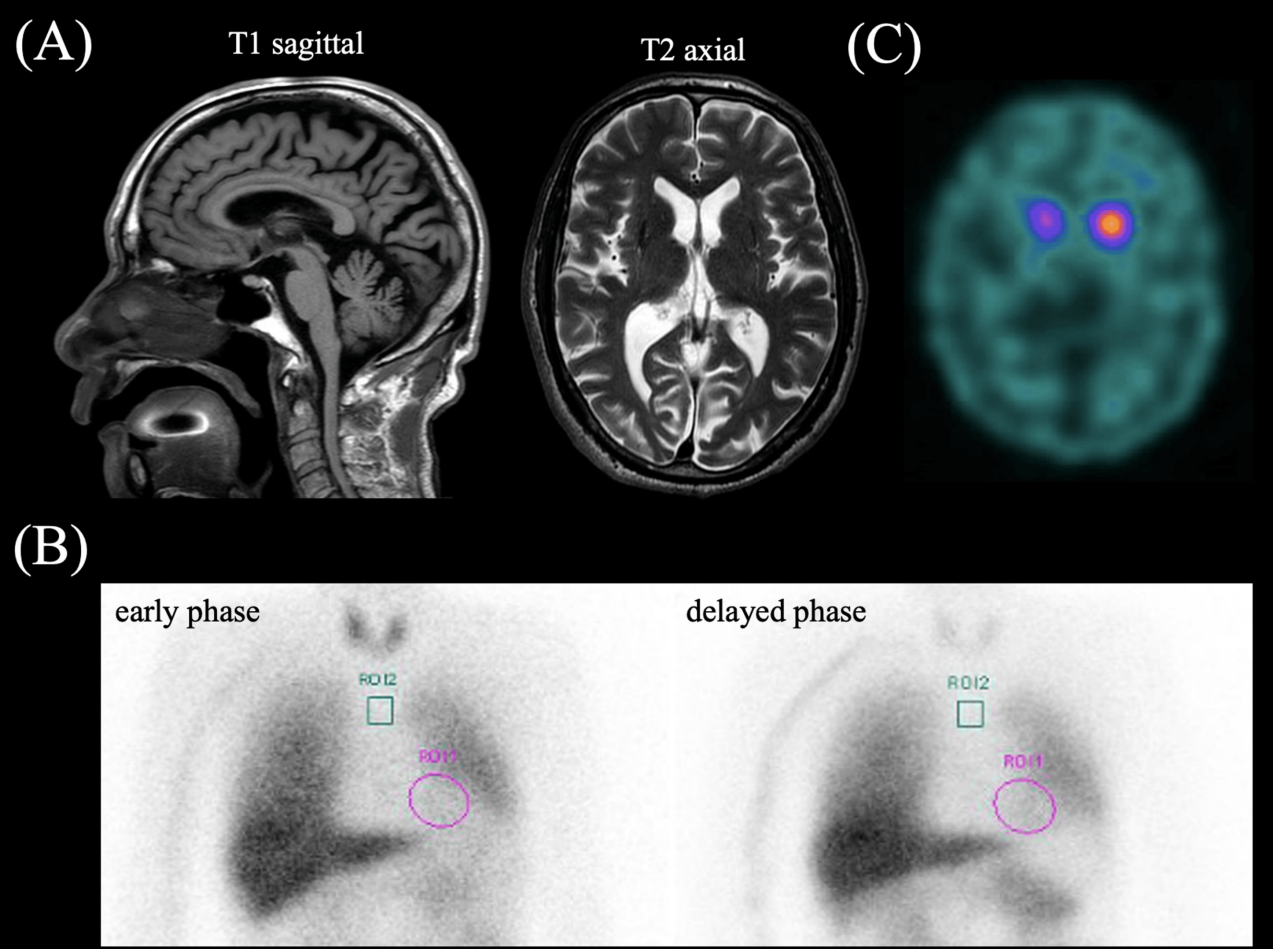
Neuroimaging findings in the patient (A) Brain magnetic resonance imaging and angiography revealed no structural abnormalities; (B) Cardiac ^123^I-metaiodobenzylguanidine scintigraphy demonstrated a reduced heart-to-mediastinum (H/M) ratio at the initial evaluation (early H/M ratio, 1.985; delayed H/M ratio, 1.647; washout rate, 50.5%). The institutional reference values were early and delayed H/M ratios >2.0 and a washout rate <34%; (C) Dopamine transporter imaging revealed bilaterally reduced specific binding ratios (right, 2.48; left, 3.10).

Electrocardiography demonstrated occasional supraventricular premature contractions, and chest radiography was unremarkable. Brain MRI and magnetic resonance angiography revealed no structural abnormalities (Figure 1A).

At the initial evaluation, ^123^I-MIBG myocardial scintigraphy demonstrated reduced cardiac uptake, with an early H/M ratio of 1.985, a delayed H/M ratio of 1.647, and a washout rate of 50.5% (Figure 1B; reference values: early and delayed H/M ratios, >2.0; washout rate, <34%). Dopamine transporter imaging revealed a bilateral reduction in the specific binding ratio (Figure 1C), indicating presynaptic dopaminergic dysfunction.

During hospitalization, the dose of levodopa/carbidopa was increased to 450 mg/day, and selegiline was titrated to 10 mg/day. A rotigotine transdermal patch was introduced and increased to 13.5 mg/day, resulting in a slight improvement in gait disturbance. The patient was discharged at the end of November 2018, i.e., after five months.

**Figure 2:**
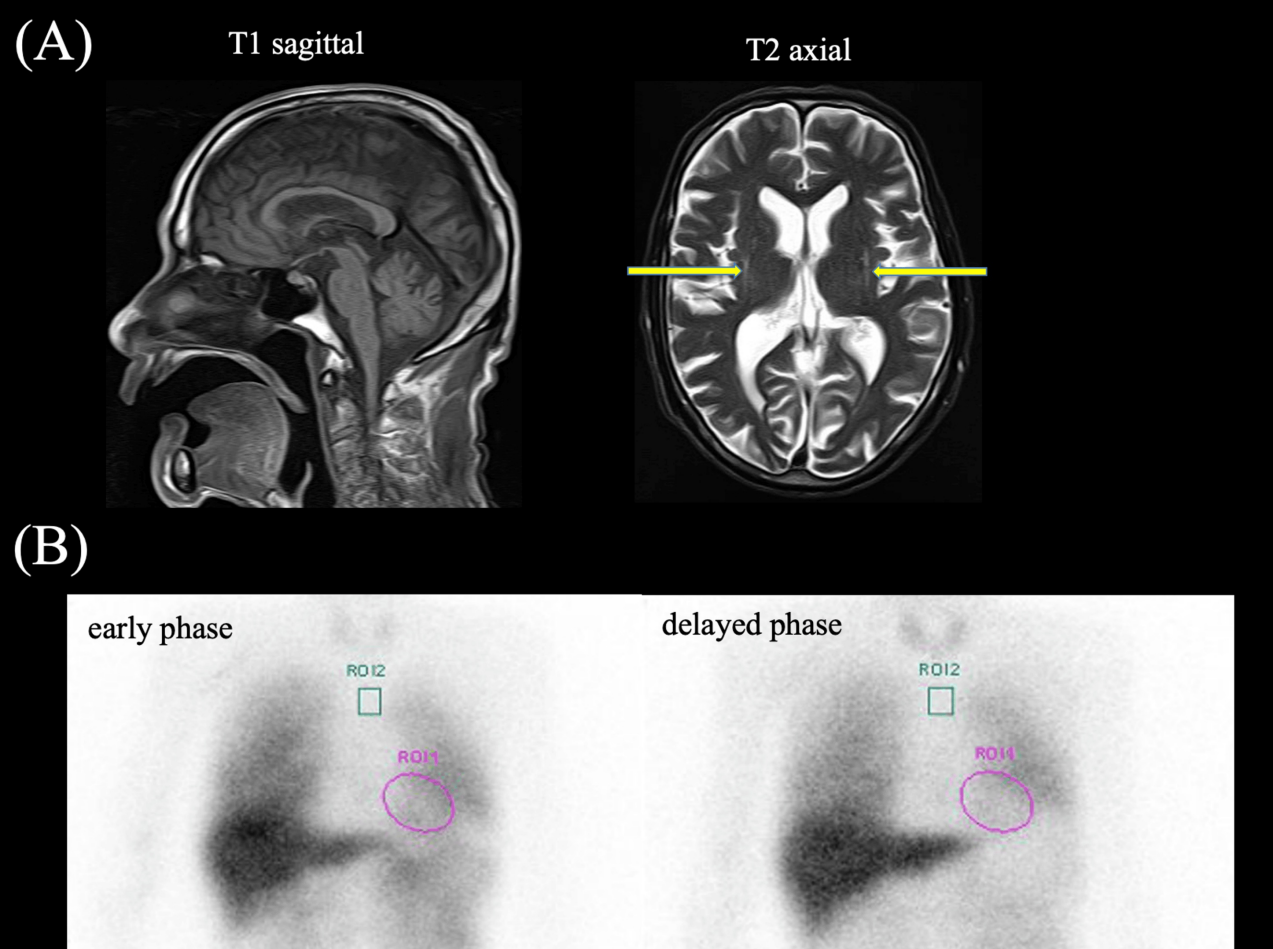
Follow-up imaging findings and changes in the cardiac sympathetic function (A) Follow-up brain magnetic resonance imaging showed hyperintense rims along the lateral margins of the bilateral putamina on T2-weighted imaging (yellow arrows), suggestive of multiple system atrophy–parkinsonian type (MSA-P); (B) After selegiline discontinuation for 28 days, repeat cardiac ^123^I-metaiodobenzylguanidine scintigraphy showed improved early and delayed H/M ratios of 2.653 and 2.032, respectively, with a washout rate of 46.0%. The institutional reference values were early and delayed H/M ratios >2.0 and a washout rate <34%.

In January 2019, i.e., after two months, he was readmitted with a 10-day history of severe constipation, abdominal pain, anorexia, and marked bilateral lower limb edema. He was diagnosed with a perforated duodenal ulcer based on imaging findings, including subdiaphragmatic free air on chest radiography and intraperitoneal free air on abdominal computed tomography, and underwent emergency surgery. His postoperative course was generally favorable, and he was transferred back to the neurology department in February. However, this episode represented a clinical turning point, after which his activities of daily living declined and more prominent MSA-related features emerged. Removal of the urinary catheter was difficult, suggesting the development of neurogenic bladder. Orthostatic hypotension was also observed, with a marked decrease in blood pressure from 114/80 mmHg in the supine position to 70/54 mmHg in the standing position, corresponding to a systolic decrease of 44 mmHg and a diastolic decrease of 26 mmHg. This finding fulfilled the criterion for autonomic dysfunction due to orthostatic hypotension. Follow-up brain MRI revealed a hyperintense rim along the lateral margin of the bilateral putamina on T2-weighted imaging, suggesting MSA-P (Figure 2A).

Together with parkinsonism, poor levodopa responsiveness, autonomic dysfunction, and characteristic MRI findings suggestive of MSA-P, the patient fulfilled the 2022 Movement Disorder Society criteria for clinically established MSA-P [2]. After obtaining informed consent, selegiline was discontinued, and repeat ^123^I-MIBG myocardial scintigraphy was performed 28 days after selegiline discontinuation. The repeat examination demonstrated improved cardiac uptake, with an early H/M ratio of 2.653, a delayed H/M ratio of 2.032, and a washout rate of 46.0% (Figure 2B; reference values: early and delayed H/M ratios, >2.0; washout rate, <34%). Compared with the initial examination, both the early and delayed H/M ratios increased above the reference threshold, although the washout rate remained elevated.

According to the Movement Disorder Society's diagnostic criteria [2], the patient fulfilled the criteria for clinically established MSA-P based on the combination of parkinsonism, autonomic dysfunction, and characteristic MRI findings. Neurological symptoms exhibited limited improvement, and the patient was transferred to a long-term care hospital after four months, i.e., in April of the same year.

## Discussion

The present report describes a patient initially diagnosed and treated for PD who was ultimately diagnosed with MSA-P based on his clinical progression and evolving imaging findings. The clinical significance of this case can be summarized in three key aspects: (1) the difficulty of early differential diagnosis, (2) the limitations of interpreting ^123^I-MIBG myocardial scintigraphy, and (3) the importance of longitudinal assessment.

Regarding the initial diagnosis, the patient presented with parkinsonism characterized by tremors, rigidity, and bradykinesia, and dopamine transporter imaging revealed a bilateral reduction in uptake. These findings were consistent with the presentation of PD. However, the patient’s response to levodopa was limited, and atypical features, such as Pisa syndrome and early postural instability, emerged during the clinical course. These findings support a diagnosis of MSA rather than PD [1]. Recent diagnostic criteria for MSA emphasize the importance of integrating motor features with autonomic dysfunction and additional supportive clinical findings [2]; the present case illustrates the clinical utility of this comprehensive diagnostic framework.

The interpretation of MIBG myocardial scintigraphy in the present case warrants careful consideration. At the initial evaluation, the H/M ratio was reduced, which was initially considered suggestive of PD, although not diagnostic. This finding may represent a diagnostic challenge as reduced cardiac MIBG uptake is typically observed in PD because of postganglionic sympathetic denervation, whereas it is relatively preserved in MSA [3-5]. However, atypical findings may occur in clinical practice, and variability in the degree of peripheral sympathetic involvement in patients with PD may contribute to these discrepancies [3-5]. Furthermore, recent reviews have emphasized that MIBG scintigraphy should be regarded as an adjunctive tool rather than a definitive diagnostic test [6].

Given that ^123^I-MIBG is taken up into presynaptic sympathetic nerve terminals via the norepinephrine transporter and subsequently stored in intraneuronal vesicles, medications that affect these processes may reduce myocardial MIBG accumulation. Previous studies have suggested that some medications, particularly those affecting presynaptic sympathetic nerve terminals and norepinephrine handling, may interfere with cardiac MIBG uptake [10]. A recent case report also described improvement in the H/M ratio after selegiline discontinuation, suggesting a potential drug-related effect on MIBG imaging [11]. In the present case, the first ^123^I-MIBG myocardial scintigraphy was performed while the patient was receiving selegiline and showed reduced cardiac MIBG uptake, with an early H/M ratio of 1.985, a delayed H/M ratio of 1.647, and a washout rate of 50.5%. After obtaining informed consent, selegiline was discontinued, and repeat ^123^I-MIBG myocardial scintigraphy was performed after a 28-day discontinuation period. The repeat examination showed improvement of the early and delayed H/M ratios to 2.653 and 2.032, respectively. These temporal findings raise the possibility that selegiline may have influenced cardiac MIBG uptake and contributed to diagnostic uncertainty. However, this interpretation remains speculative because it is based on a single observation in one patient and supported by limited evidence. Therefore, a causal relationship between selegiline use and changes in MIBG uptake cannot be established from this case alone, and MIBG findings obtained during selegiline treatment should be interpreted with caution.

Neuroimaging findings also played a decisive role in the diagnosis of our patient. Follow-up MRI revealed a hyperintense rim along the lateral margin of the bilateral putamina on T2-weighted imaging, a feature considered relatively specific for MSA-P and useful for differentiating it from PD [7].

Together, these imaging findings contributed to the diagnostic reclassification of our case and provided a key objective basis for this process. The present case highlights the potentially reversible nature of MIBG abnormalities and underscores the need for MIBG findings during selegiline treatment to be cautiously interpreted in the differential diagnosis of PD and MSA.

The follow-up MRI findings, together with the subsequent emergence of autonomic dysfunction and limited levodopa responsiveness, provided an objective basis for reclassifying the diagnosis from PD to MSA-P. Although the initial clinical and imaging findings appeared to support PD, the longitudinal clinical course and characteristic putaminal abnormalities ultimately favored MSA-P. This case suggests that a single-time-point assessment may be insufficient in the early stage of parkinsonism, particularly when atypical features emerge during follow-up. Repeated clinical and imaging evaluations are therefore essential for distinguishing PD from MSA. In addition, the improvement in cardiac MIBG uptake after selegiline discontinuation raises the possibility that selegiline treatment may have contributed to the initially abnormal MIBG findings.

## Conclusions

This case illustrates the diagnostic challenge of differentiating PD from MSA-P in the early stage of parkinsonism. Although the initial clinical and imaging findings supported a diagnosis of PD, the subsequent emergence of autonomic dysfunction, limited levodopa responsiveness, and characteristic putaminal MRI abnormalities led to diagnostic reclassification as MSA-P. The improvement in cardiac MIBG uptake after selegiline discontinuation suggests that medication effects should be considered when interpreting MIBG scintigraphy findings. However, this observation should be interpreted cautiously because it is based on a single case. Repeated clinical and imaging assessments are essential when atypical features emerge during follow-up. Longitudinal multimodal evaluation may improve diagnostic accuracy in patients with evolving parkinsonian syndromes.
